# Harmonic Ratio Analysis in Magnetic Particle Imaging Enables Differentiation of Malignant and Benign Human Breast Tissues: A Feasibility Study

**DOI:** 10.3390/bioengineering13020183

**Published:** 2026-02-04

**Authors:** Hongyu Yang, Haoran Zhang, Yiyin Zhang, Yixiang Zhou, Xinmiao Qu, Xun Zhang, Ke Li, Hanfu Shi, Hui Lin, Shu Wang, Zeyu Zhang

**Affiliations:** 1 Key Laboratory of Big Data-Based Precision Medicine of Ministry of Industry and Information Technology, School of Engineering Medicine, Beihang University, Beijing 100191, China; 2 School of Biological Science and Medical Engineering, Beihang University, Beijing 100191, China; 3 Breast Center, Peking University People’s Hospital, Peking University, Beijing 100044, China; dr_yiyin_zhang@hotmail.com (Y.Z.);; 4 Academy of Macroeconomic Research National Development and Reform Commission of People’s Republic of China, Beijing 100038, China; 5 Department of General Surgery, Sir Run Run Shaw Hospital, School of Medicine, Zhejiang University, Hangzhou 310018, China; 6 College of Biomedical Engineering and Instrument Science, Zhejiang University, Hangzhou 310027, China

**Keywords:** Magnetic Particle Imaging (MPI), breast cancer, harmonic ratio, magnetic nanoparticles, lymph nodes, tissue characterization

## Abstract

Accurate intraoperative differentiation between malignant and benign breast tissues, particularly the assessment of lymph node status and tumor margins, is critical for surgical decision-making and prognosis. Traditional histopathological methods, such as frozen section analysis, are time-consuming and labor-intensive. Magnetic Particle Imaging (MPI) is a novel, radiation-free modality that senses the microenvironmental properties of tissues through the dynamic response of magnetic tracers. In this study, we propose a diagnostic method utilizing the higher-order harmonic response of magnetic nanoparticles. Various ex vivo breast tissue samples were immersed in Synomag-50 nanoparticles. Using a custom-built MPI spectrometer (5 kHz excitation, 9 mT amplitude) operating in spectroscopic mode, we implemented a rapid acquisition protocol in which each sample was measured 10 times, with 0.1 s per cycle. We analyzed the magnetic response spectrum and calculated the ratio of the third to the fifth harmonic ($H_{3}/H_{5}$). Histological analysis confirmed the effective infiltration of MNPs into the interstitial spaces. The repeated measurement data demonstrated high stability. A distinct stepwise increase in harmonic ratios was observed from normal tissue to tumor-adjacent tissue and finally to malignant tumors. Specifically, malignant samples showed ratios that generally exceeded 2.2, whereas benign samples remained below 2.0. These preliminary findings suggest that the harmonic ratio could serve as a sensitive biomarker reflecting the microenvironmental constraints associated with malignancy. This study validates the feasibility of utilizing MPI signal harmonics as a quantitative metric with rapid signal acquisition capabilities for differentiating benign and malignant lymph nodes.

## 1. Introduction

Breast cancer is the most common malignant tumor among women worldwide, posing a significant threat to health and life [1,2]. Accurate staging of breast cancer, particularly the assessment of axillary lymph nodes and the determination of tumor margins, is crucial for formulating effective treatment plans and enhancing patient survival rates [3]. Currently, clinical practice mainly relies on preoperative imaging techniques, such as mammography, computed tomography (CT), and magnetic resonance imaging (MRI) [4,5,6]. Although these imaging modalities provide important structural information in advance, they often struggle to facilitate real-time qualitative diagnosis during surgery and may lack sufficient specificity [7]. Furthermore, the gold standard for intraoperative assessment—frozen-section histopathology—requires considerable processing time, with each sample taking 20 to 30 min, and its results can be affected by sampling errors [8]. Hence, there is an urgent need for a rapid, accurate, and automated technology to effectively differentiate malignant tissue from benign tissue during surgery [9,10,11].

While Magnetic Resonance Imaging (MRI) remains the clinical gold standard for soft-tissue evaluation with contrast agents such as gadolinium chelates, it primarily relies on detecting the indirect effects of these agents on proton relaxation times. In contrast, MPI offers a distinct physical advantage: it directly detects the nonlinear magnetization response of superparamagnetic iron oxide nanoparticles (SPIONs) [12,13]. Since biological tissues are diamagnetic and do not generate a nonlinear magnetic response, MPI benefits from a ‘zero-background’ signal environment [14]. This characteristic enables high-contrast visualization and accurate quantification of the tracer distribution without interference from the anatomical tissue background [15,16].

To understand the mechanism of tissue differentiation, it is critical to distinguish between the two primary relaxation modes of SPIONs. While standard MPI imaging often relies on Néel relaxation for the quantitative determination of particle concentration independent of the environment, the sensing capability utilized in this study depends on Brownian relaxation. Unlike Néel relaxation, which is internal to the crystal structure, Brownian relaxation involves the physical rotation of the entire hydrodynamic volume, rendering it highly sensitive to the microenvironmental viscosity and steric constraints. Consequently, this study employs a Magnetic Particle Spectroscopy (MPS) approach—analyzing the harmonic spectra generated specifically by inhibited Brownian motion—to characterize the relaxation dynamics of nanoparticles within the tissue matrix, distinct from conventional spatial imaging reconstruction.

There are notable differences in the microenvironment and biomechanical characteristics between tumor and normal tissues. The malignant transformation process is typically accompanied by fibrosis, increased interstitial fluid pressure, and heightened collagen cross-linking, leading to significantly higher local viscosity and stiffness compared to benign tissue [17,18]. Consequently, when SPIONs are located within breast tissue, the signal spectrum changes detected by the MPI system will be directly related to alterations in the tissue microenvironment [15,19,20].

In this study, we aim to validate this hypothesis using a miniaturized, low-frequency MPI system. By measuring the harmonic ratios of ex vivo human breast tissues and lymph nodes loaded with nanoparticles, we investigate whether specific signal signatures ($H_{3}/H_{5}$ ratio) can effectively differentiate benign from malignant pathologies. This work provides a foundation for the subsequent in vivo differentiation of benign and malignant breast tissue.

## 2. Materials and Methods

### 2.1. Theoretical Framework: Harmonic Generation

The fundamental signal in MPI arises from the non-linear magnetization curve of superparamagnetic nanoparticles [13,21]. When exposed to an alternating magnetic field H(t), the magnetization *M* of the particle ensemble follows the Langevin function. For a mono-dispersed system, this is described as follows:

(1) $$M\left(H\right)=m\cdot c\cdot L\left(\xi \right)\;\mathrm{with}\;\xi =\frac{\mu_{0}m∥H∥}{k_{B}T}$$

where *m* is the magnetic moment of a single particle, *c* is the concentration of the particles, $\mu_{0}$ is the vacuum permeability, $k_{B}$ is the Boltzmann constant, and *T* is the absolute temperature. The Langevin function L(ξ) is defined as follows:

(2) $$L\left(\xi \right)=\coth\left(\xi \right)-\frac{1}{\xi }$$

It is important to note that Equation (1) describes the magnetization of an ideal mono-dispersed particle system. In practice, the magnetic nanoparticles (Synomag-50) exhibit a particle size distribution, typically following a log-normal law. While the mono-dispersed model suffices to illustrate the fundamental mechanism of harmonic generation in this feasibility study, precise quantification in future work should account for the polydisperse granulometry of the particle ensemble. It is important to note that the Langevin function describes the magnetization of particles in thermodynamic equilibrium. At the excitation frequency of 5 kHz used in this study, this model serves as a useful approximation. More rigorous solutions involving the Fokker–Planck equation would be required to fully model the dynamic non-equilibrium relaxation effects; however, the equilibrium approximation is sufficient to illustrate the fundamental harmonic generation mechanism relevant to this study. Due to the non-linearity of the Langevin function L(·), the time-dependent magnetization contains the fundamental excitation frequency $f_{0}$ and a series of odd harmonics ($3f_{0},5f_{0},\ldots$) [22].

To generate a signal, a spatially uniform, time-varying excitation field ($A_{D}\sin\left(2\pi f_{0}t\right)$) is used, where $A_{D}$ represents the amplitude of the drive field (9mT), and $f_{0}$ is the excitation frequency (5kHz). This excitation causes the magnetization of the SPIONs to oscillate rapidly. According to Faraday’s Law and the reciprocity principle, the change in particle magnetization induces a voltage u(t) in the receive coil:

(3) $$u\left(t\right)=-\mu_{0}\int_{V}p\left(r\right)\cdot \frac{\partial M(r,t)}{\partial t}\;dV$$

where p(r) represents the receive coil sensitivity profile, and *V* is the sample volume [13].

**Figure 1:**
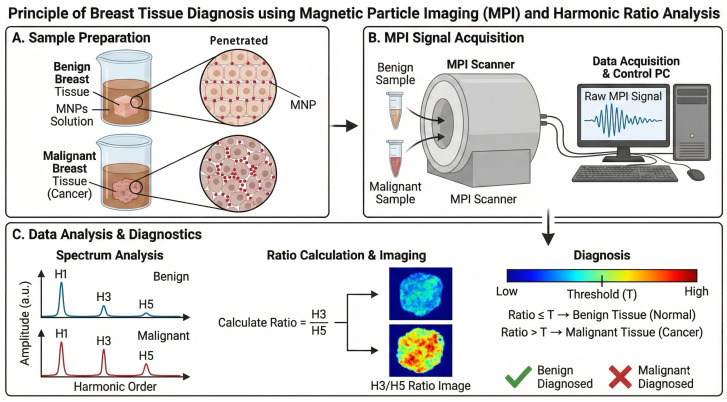
Schematic of the MPI-based breast tissue diagnosis workflow. (**A**) Sample preparation: Benign and malignant tissues are immersed in a magnetic particle solution after being excised, allowing the magnetic particles to diffuse into the tissues. (**B**) Signal acquisition: Once the tissue samples are removed, they are placed in the MPI system to capture the corresponding magnetic response signals. (**C**) Data analysis: After performing frequency domain analysis on the magnetic response signals, the harmonic intensities are extracted and the $H_{3}/H_{5}$ harmonic ratios are calculated to determine the benign or malignant nature of the tissues based on the magnitude of the harmonic ratios.

The relationship between harmonic ratios and environmental properties is well-established in magnetic particle spectroscopy [23]. Specifically, increased microenvironmental viscosity or stiffness hinders the Brownian rotation of nanoparticles, leading to increased phase lag and a shift in the harmonic spectrum [19,24]. In this study, we empirically utilize the $H_{3}/H_{5}$ ratio as a surrogate marker for these biophysical changes. The principle and process of the experiment are shown in Figure 1.

### 2.2. MPI System Setup

A miniaturized Magnetic Particle Imaging (MPI) system was utilized in this study. The system architecture and its tomographic imaging capabilities have been fully characterized in our previous work [25]. However, for the specific purpose of quantitative tissue characterization in this study, the system was operated to analyze the harmonic signal spectra from the sensitive region, rather than to reconstruct spatial images. This approach leverages the high sensitivity of the MPI hardware to detect microenvironmental changes via the particle’s dynamic response [26]. The system architecture employs a unique cancellation strategy that combines a handheld detection unit with a stationary external compensation unit, effectively suppressing the fundamental excitation signal while maintaining portability.

#### 2.2.1. Coil Configuration and Design

The handheld unit, designed for single-handed operation with a height of 130mm, houses an excitation coil and a nested receive coil. This unit serves to excite superparamagnetic iron oxide nanoparticles (SPIONs) and detect their nonlinear responses. The external compensation unit is stationary and table-mounted, consisting of an external excitation coil and a compensation coil. It is equipped with a mechanical fine-tuning stage that allows for relative displacement adjustments (*x*-axis: 6mm; *z*-axis: 5mm) to achieve precise magnetic flux balancing.

To eliminate the direct feedthrough signal, the excitation coils in both units are wound in the same direction and connected in series, whereas the receiver coil and the compensation coil are wound in opposite directions and connected in series. The specific geometric and electrical parameters of the coils are detailed in Table 1.

#### 2.2.2. Signal Acquisition and Processing Chain

**Figure 2:**
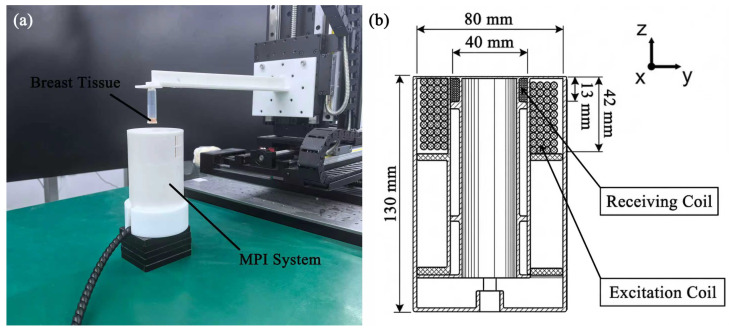
Experimental setup of the miniaturized MPI system. (**a**) Photograph of the system showing the cantilever sample holder positioning the breast tissue sample into the detection module. (**b**) Cross-sectional schematic of the coil structure with dimensions in millimeters. The probe consists of an outer excitation coil and an inner receiving coil, featuring a 40 mm bore diameter and an outer diameter of 80 mm.

The system hardware is composed of three functional modules: the excitation module, the receive module, and the signal acquisition module (Figure 2).

The excitation module drives the excitation coil with a sinusoidal current of 20 A at 5 kHz, generating an alternating magnetic field of 9 mT. A high-precision current sensor monitors current stability in real time to ensure that the tissue samples remain within a sufficiently strong magnetic field throughout the detection process. The receive module captures the magnetic response signals generated by the SPIOs within the tissue samples; these signals are amplified by a factor of 10 to enhance the signal-to-noise ratio (SNR). Finally, the data acquisition module records the signals from the receive module at a sampling rate of 1 MS/s. This high sampling rate ensures accurate capture of higher-order harmonics, which is crucial for calculating harmonic ratios.

### 2.3. Sample Collection and Protocol

Human breast tissue samples were obtained from patients undergoing mastectomy or lumpectomy. To strictly preserve the native biomechanical properties of the extracellular matrix, a fresh tissue protocol was implemented. All samples were processed ex vivo immediately without chemical fixation or freezing. Prior to measurement, samples were gently rinsed with saline to remove surface blood. Subsequently, the samples were incubated in the Synomag-50 solution for a standardized period of 20 min at a controlled temperature of 27 °C. This duration was determined based on preliminary tests to ensure sufficient signal intensity for detection. We acknowledge that passive diffusion does not guarantee uniform particle concentration across heterogeneous tissue types; however, this duration provided adequate SNR for the harmonic analysis in this feasibility study. Measurements were performed immediately following incubation to minimize tissue degradation, ensuring that all data were acquired from fresh, biologically active specimens. We explicitly acknowledge that the number of unique biological specimens in this pilot cohort is limited. Therefore, this study is designed to evaluate the technical feasibility of the sensing principle, rather than to establish statistical diagnostic sensitivity or specificity.

A diverse set of tissue samples was collected and categorized into seven groups based on standard histopathological evaluation:

1. Normal Control: Healthy breast tissue (Normal-1, Normal-2).
2. Benign Lesions: Benign Lymph Nodes (BLNs).
3. Malignant Tumors: Invasive Breast Cancer (IBC), Mucinous Carcinoma (MC), and Metastatic Lymph Nodes (MLNs).
4. Adjacent Tissue: Adjacent Normal Tissue (ANT), collected from the distinct margin between tumor and healthy tissue.

To evaluate signal stability and instrument repeatability, a repeated measurement protocol was implemented. Each unique tissue specimen was measured consecutively 10 times ($n_{tech}=10$). The data acquisition time for a single measurement was 0.1 s, and the final harmonic ratio for each sample was derived by averaging the data from these 10 independent acquisition cycles. It should be noted that this work is presented as a preliminary feasibility study demonstrating proof of concept. Sample collection is currently ongoing to expand the cohort for large-scale clinical validation in future studies.

### 2.4. Magnetic Nanoparticles

In this study, we utilized Synomag-50 particles (Micromod Partikeltechnologie GmbH, Germany). Structurally, these are “nanoflower-shaped” multi-core particles consisting of densely packed iron oxide cores (crystallite size ∼5–10 nm) embedded in a dextran matrix, with a total hydrodynamic diameter of approximately 50 nm [27]. This specific multi-core architecture is critical for the study’s objective. Unlike single-domain crystals of 50 nm, which would exceed the superparamagnetic limit (∼25 nm for magnetite) and exhibit ferromagnetic blocking behavior, or isolated small single cores dominated by the Néel mechanism, the nanoflower structure maintains the superparamagnetic nature of the individual cores while ensuring the entire cluster undergoes Brownian rotation. This unique property maximizes sensitivity to microenvironmental stiffness while preserving high specific loss power (SLP), making them highly suitable for this application [28,29].

### 2.5. Data Processing

Raw time-domain signals acquired from the system were processed. Prior to the Fast Fourier Transform (FFT), a Hanning window was applied to the data to mitigate spectral leakage. From the resulting frequency spectrum, the peak amplitudes of the third harmonic ($H_{3}$ at 15kHz) and the fifth harmonic ($H_{5}$ at 25kHz) were extracted. The spectral resolution was determined to be 10Hz, corresponding to the acquisition time of 0.1s. Since the excitation and harmonic frequencies (5kHz, 15kHz, 25kHz) are integer multiples of the frequency resolution, we utilized the magnitude of the FFT bin directly at the corresponding harmonic frequencies to determine the signal amplitude. The diagnostic metric, *R*, for each sample was calculated as the ratio $R=H_{3}/H_{5}$.

## 3. Results

### 3.1. Verification of Nanoparticle Infiltration and Distribution

To ensure the reliability of the harmonic ratio analysis ($H_{3}/H_{5}$), we first validated that the iron content in our biological samples falls within the effective detection range of the MPI system. According to the sensitivity characterization of the handheld MPI device used in this study, the system exhibits high detection sensitivity, with a lower detection limit of approximately 50ng of iron, maintaining a high linearity ($R^{2}>0.99$) across the concentration gradient. In our experiments, the raw signal intensities acquired from the incubated tissue samples were consistently significantly higher than the signal baseline established at the 50ng threshold. This confirms that the observed harmonic signals possess a sufficient SNR. Consequently, the calculated $H_{3}/H_{5}$ ratios are statistically robust and reflective of the particles’ magnetoviscous behavior in the biological environment, rather than artifacts arising from low-concentration noise or detection instability. Prior to signal analysis, histological validation was conducted to ensure that the acquired MPI signals accurately reflect the internal characteristics of the breast tissue rather than surface artifacts. Representative tissue sections (Normal-1, ANT, and IBC) were subjected to Prussian Blue staining to visualize the spatial distribution of the iron oxide-based Synomag-50 nanoparticles.

**Figure 3:**
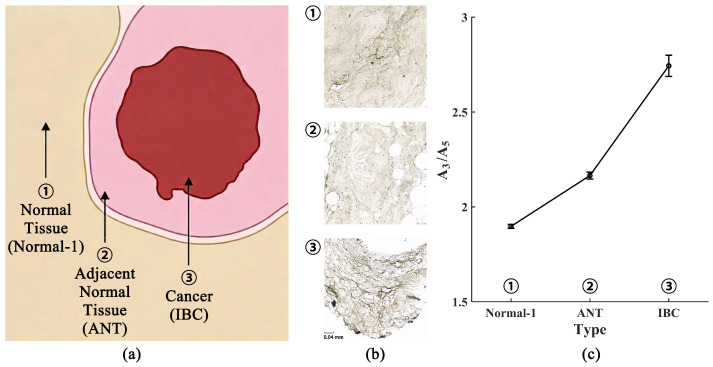
Comprehensive harmonic ratio comparison. (**a**) Schematic illustration showing the spatial distribution of the sampling sites: ① Normal Tissue (Normal-1), representing healthy tissue distinct from the tumor; ② Adjacent Normal Tissue (ANT), collected from the tumor margin/peritumoral region; and ③ Invasive Breast Cancer (IBC), representing the tumor core. (**b**) Histological images of the representative tissue samples. (**c**) Line graph comparing the mean $H_{3}/H_{5}$ harmonic ratios across all tissue groups (n=10 scans per sample). Malignant tissues exhibit markedly higher ratios compared to normal tissues and benign lymph nodes.

Microscopic examination revealed successful and pervasive infiltration of nanoparticles into the tissue samples. As shown in the histological results (Figure 3), distinct blue-stained regions identifying iron deposits were observed distributed within the interstitial spaces and stromal matrix of the tissues. Crucially, the particles did not merely accumulate on the surface but had passively diffused deep into the extracellular environment. This confirms that the subsequent harmonic signals originated from nanoparticles interacting directly with the internal tissue microstructure. The effective incorporation of superparamagnetic iron oxide nanoparticles ensures that variations in harmonic response can be attributed to specific physical constraints imposed on the nanoparticles by the internal tissue microenvironment.

Furthermore, the harmonic ratio results presented in Figure 3c indicate distinct differences among the various breast tissue types. Specifically, as the sampling location approached the tumor core, a monotonic increase in the harmonic ratio was observed, rising from 1.898 to 2.744.

### 3.2. Quantitative Analysis of Harmonic Ratios

**Figure 4:**
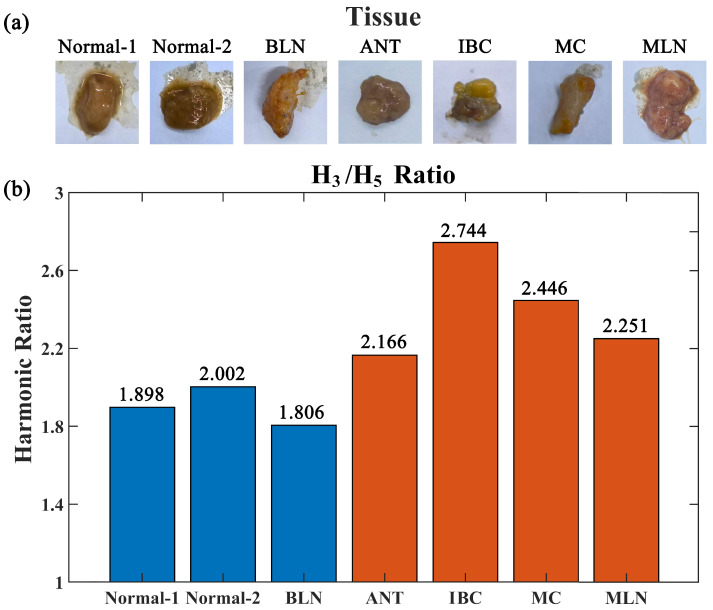
Detailed harmonic ratio analysis across samples. (**a**) Gross photographs of the representative tissue samples. (**b**) Bar chart comparing the mean $H_{3}/H_{5}$ harmonic ratios across all tissue groups (n = 10 scans per sample). Blue and orange respectively represent benign and malignant tissues. Malignant tissues (IBC, MC, MLN) exhibit markedly higher ratios compared to normal tissues and benign lymph nodes.

The harmonic ratios ($H_{3}/H_{5}$) for the different tissue groups, calculated based on the rapid acquisition protocol (averaging 10 scans per sample), are illustrated in Figure 4. The quantitative data reveal a distinct stratification of signal characteristics that correlates strongly with the pathological classification of the samples.

#### 3.2.1. Baseline Signature of Benign and Normal Tissues

The baseline group, comprising healthy breast tissue and benign lesions, exhibited characteristic harmonic ratios that were both stable and relatively low. Specifically, the two normal breast tissue samples (Normal-1 and Normal-2) yielded ratios of 1.898 and 2.002, respectively. Similarly, the BLN sample exhibited the lowest ratio across the entire dataset, at 1.806.

These values suggest a distinct interaction pattern of the nanoparticles within healthy tissue structures. In these non-malignant environments, the $H_{3}/H_{5}$ ratios clustered at levels below approximately 2.0. The consistency of these low ratios implies that under healthy or benign conditions, the tissue microenvironment—likely characterized by a looser matrix network and normal cellular density—imposes similar and minimal constraints on the magnetic response of the nanoparticles. This establishes a robust baseline for healthy tissue, against which pathological changes can be contrasted.

#### 3.2.2. Elevated Response in Malignant Pathologies

In stark contrast to the baseline group, all samples histopathologically confirmed as malignant exhibited significantly elevated harmonic ratios. The IBC sample yielded the highest harmonic response, reaching a peak ratio of 2.744. This represents a substantial increase of approximately 45% relative to the baseline normal tissue (Normal-1). The MC sample also presented a markedly high ratio of 2.446.

This pervasive elevation across distinct cancer subtypes indicates that malignancy induces fundamental alterations in the physical properties of the tissue, thereby significantly modulating the nonlinear magnetization dynamics of SPIONs. While specific biological drivers may vary among subtypes, the consistent attenuation of the fifth harmonic relative to the third (resulting in an elevated ratio) suggests that the malignant microenvironment imposes a greater degree of physical constraint on the nanoparticles. This constraint likely stems from the dense, disordered, and stiff nature of the tumor stroma, as well as the high cellular density typical of these cancerous tissues.

#### 3.2.3. Differentiation of Lymph Nodes

A pivotal finding of this study is the clear diagnostic separation observed between BLN and MLN. As previously noted, the BLN samples displayed a baseline ratio of 1.806. In contrast, the MLN samples, representing nodes compromised by metastatic invasion, presented a markedly distinct ratio of 2.251.

The differential of 0.445 between benign and metastatic nodes is of particular significance. It indicates that when cancer cells infiltrate lymph nodes, the resulting tumor cell proliferation and stromal remodeling sufficiently alter the internal physical properties of the node to shift its MPI signal signature out of the benign range. This clear distinction validates the potential of the $H_{3}/H_{5}$ ratio as a rapid, quantitative biomarker for intraoperative sentinel lymph node assessment, offering a distinct separation between healthy and involved nodes within this specific sample set.

#### 3.2.4. Intermediate Signals in Tumor-Adjacent Tissue

The analysis of the ANT yielded a particularly revealing result. The ANT samples exhibited an intermediate harmonic ratio of 2.166. This value is significantly elevated compared to pure normal tissue (range: 1.89–2.00) yet remains distinctly lower than the definitive tumor core (IBC: 2.744).

Although ANT is histologically classified as non-malignant marginal tissue, its elevated MPI signal suggests that the method is sensitive to the peri-tumoral “field effect”. Tissues immediately adjacent to tumors often undergo subtle microstructural and molecular alterations—such as extracellular matrix stiffening or pre-cancerous stromal activation—long before overt morphological changes manifest. The fact that the harmonic ratio captures this gradient (Normal < ANT < Cancer) indicates that MPI is sensing progressive alterations in the tissue microenvironment. This sensitivity to the transitional zone underscores the method’s potential utility in precisely delineating tumor margins, offering the capability to detect risk zones that may be radiologically occult in conventional structural imaging.

In conclusion, the experimental results demonstrate a robust correlation between the $H_{3}/H_{5}$ and histopathology. In this pilot cohort, we observed a trend where benign samples generally exhibited lower ratios compared to malignant samples, with adjacent tissues showing intermediate values. While preliminary empirical ranges were observed (e.g., benign samples falling below 2.0 in this dataset), these values should not be interpreted as validated diagnostic zones given the limited sample size and existence of transitional values. This validates the feasibility of utilizing this magnetic signal signature for rapid tissue characterization. It should be noted that the diagnostic thresholds presented here are empirical observations derived from this pilot dataset. We acknowledge the existence of borderline cases, particularly in transitional zones. Establishing robust clinical cut-off values with defined sensitivity and specificity will require a larger cohort study and Receiver Operating Characteristic (ROC) curve analysis.

## 4. Discussion

### 4.1. Microenvironmental Constraints and Signal Distortion

Malignancy is characterized by the extracellular matrix (ECM) densification driven by cancer cell proliferation and the desmoplastic reaction (i.e., the formation of fibrous connective tissue). This structural densification significantly restricts the physical volume available for the Brownian rotation of the 50nm nanoparticles. Under a 5kHz alternating magnetic field, particles within this confined environment are unable to reorient sufficiently fast to track the excitation frequency, resulting in a suppressed magnetization response. In signal processing terminology, this suppression functions as a physical low-pass filter; it disproportionately attenuates higher-frequency components relative to lower-frequency ones, thereby driving an increase in the harmonic ratio [19].

It is also crucial to distinguish the effects of particle concentration from microenvironmental constraints. We acknowledge that nanoparticle uptake may vary between tissue types due to differences in vascular permeability and interstitial retention. However, a fundamental advantage of the harmonic ratio method is its concentration independence. According to the Langevin theory (Equation (1)), the magnetization response is linearly proportional to the particle concentration (*c*). Consequently, variations in concentration scale the amplitudes of all harmonics ($H_{3}$ and $H_{5}$) equally. The ratio $R=H_{3}/H_{5}$, therefore, acts as a self-normalized metric, effectively canceling out the concentration factor. While the harmonic ratio is theoretically concentration-independent based on the equilibrium Langevin model, we acknowledge that in a realistic biological environment, extremely low local concentrations could lead to poor SNR, thereby affecting the stability of the ratio calculation. Furthermore, particle aggregation or varying degrees of immobilization within the tissue matrix could introduce complex relaxation dynamics not fully captured by the simple concentration-independent model. Future studies should include direct iron quantification to correlate loading efficiency with signal stability. Consequently, the concentration independence claimed here remains a theoretical advantage that requires further empirical validation rather than a proven outcome in this pilot cohort.

Comparison with In Vitro Models: While emerging technologies such as organ-on-a-chip and 3D bioprinting offer controlled environments for studying tumor mechanobiology, they remain simplified models of the complex in vivo heterogeneity. In this study, we prioritized the use of fresh patient-derived tissues to capture the authentic, chaotic microarchitecture of human breast cancer. However, we acknowledge that future standardization of this MPI method could benefit from validation using microfluidic tumor spheroids to strictly control variables like collagen density and interstitial pressure.

While our data strongly supports the link between harmonic distortion and malignancy, we acknowledge that direct mechanical testing was not performed in this study to quantify the specific stiffness values of the samples. However, the observed elevation in the $H_{3}/H_{5}$ ratio aligns with established literature describing the distinct biomechanical properties of breast carcinoma [17,18]. While our results are consistent with the hypothesis that malignancy-induced ECM densification restricts Brownian rotation, we clarify that without paired mechanical testing, the harmonic ratio should be interpreted as a composite metric of microenvironmental mobility constraints rather than a direct measure of tissue stiffness. Future studies will incorporate independent mechanical validation to correlate harmonic ratios directly with tissue Young’s modulus.

### 4.2. The “Field Effect” in Adjacent Tissue

A notable finding is the observation of an intermediate ratio (2.166) in the Adjacent Normal Tissue (ANT), situated between that of healthy tissue and the tumor core (Figure 4). Histologically, ANT typically presents as normal tissue under standard Hematoxylin and Eosin (H&E) staining. However, the elevated MPI signal suggests that our method is sensitive to the “field effect”—molecular and microstructural alterations occurring in the peri-tumoral stroma, such as increased collagen deposition or pre-cancerous stiffening. This sensitivity underscores the potential of MPI for the precise assessment of tumor margins, offering the capability to identify risk zones that may be overlooked during macroscopic inspection.

### 4.3. Clinical Implications

The primary limitation of this study is the small biological sample size. With only a limited number of unique specimens per class, the threshold values reported here should be interpreted as preliminary empirical observations rather than validated diagnostic cut-offs. Large-scale recruitment is currently underway to address this limitation. We describe the method as “rapid” specifically referring to the signal acquisition time (0.1 s). We acknowledge that the passive diffusion of nanoparticles into excised tissue introduces a significant time cost (20 min in this protocol), which dominates the total time-to-decision. Furthermore, we recognize that in this ex vivo immersion model, penetration depth varies with tissue thickness and stromal density—factors not systematically characterized in this study. Therefore, the current “dip-and-measure” workflow serves only as an experimental model, whereas future clinical translation would necessitate bypassing this diffusion bottleneck to fully leverage the real-time detection capabilities of MPI. In this ex vivo study, samples were incubated for 20 min. While this is comparable to the processing time of frozen sections, future in vivo applications could bypass this bottleneck through direct local injection or topical administration of tracers prior to excision, thereby fully leveraging the real-time detection capabilities of MPI. The current study demonstrates short-term technical signal stability under controlled experimental conditions. We explicitly acknowledge that this study did not evaluate realistic sources of clinical variability, such as day-to-day drift, coil/sample repositioning errors, or inter-operator inconsistency. Consequently, the robustness of the system for routine surgical workflows remains to be validated in future translational studies. Future translational work will prioritize a comprehensive reproducibility analysis to validate the system’s robustness for routine surgical use. We envision two potential clinical workflows for this technology. First, for intraoperative margin assessment, tracers could be administered systemically or locally prior to surgery. The handheld probe would then be used to scan the excision cavity in real-time, functioning similarly to a gamma probe in sentinel lymph node biopsy, but detecting magnetic harmonics instead of radiation. Alternatively, for ex vivo rapid assessment, excised tissue margins could be measured immediately using a “dip-and-measure” protocol (as modeled in this study) or via automated surface scanning, providing feedback to the surgeon within minutes. Furthermore, for successful in vivo translation, the granulometry and stability of the nanoparticles must be rigorously evaluated. The presence of large aggregates or blocked particles could pose physiological risks, such as thromboembolism. Future preclinical trials will therefore include detailed particle size distribution analysis to ensure hemodynamic safety.

## 5. Conclusions

In this study, we successfully demonstrated the feasibility of using Magnetic Particle Imaging to characterize breast tissues and lymph nodes based on their harmonic response. By employing a rapid 0.1 s acquisition protocol and analyzing the $H_{3}/H_{5}$ ratio of Synomag-50 nanoparticles, we observed a distinct quantitative stratification between benign and malignant samples. The results indicate that the restrictive microenvironment of malignant tissues significantly alters the relaxation dynamics of magnetic nanoparticles, providing a measurable signal contrast. With its high sensitivity, rapid readout, and lack of ionizing radiation, MPI harmonic analysis holds significant promise as a novel tool for intraoperative margin assessment and lymph node staging.

## Figures and Tables

**Table 1:** Parameters of the MPI Coils in this study.

Parameter	Excitation Coil	Ext. Excitation Coil	Receiving Coil	Compensation Coil
Turns (N)	40	40	120	120
Layers	4	4	6	4
Inner Diameter (mm)	46	56	33	34
Outer Diameter (mm)	80	90	40	40
Wire Section (${\mathrm{mm}}^{2}$)	6.28	6.28	0.16	0.16
Inductance (μH)	63.4	83.5	567.4	477.8

## Data Availability

The data presented in this study are available on request from the corresponding author due to the ongoing collaborative nature of the project with the hospital.
